# COVID-19 health inequities and association with mechanical ventilation and prolonged length of stay at an urban safety-net health system in Chicago

**DOI:** 10.1371/journal.pone.0258243

**Published:** 2021-10-13

**Authors:** Jacquelyn Jacobs, Amy K. Johnson, Arianna Boshara, Bijou Hunt, Christina Khouri, Javier Cruz, Nancy Glick

**Affiliations:** 1 Sinai Urban Health Institute, Sinai Health System, Chicago, Illinois, United States of America; 2 Ann & Robert H. Lurie Children’s Hospital of Chicago, Chicago, Illinois, United States of America; 3 Sinai Infectious Disease Center, Chicago, Illinois, United States of America; 4 Chicago Medical School at Rosalind Franklin University, North Chicago, Illinois, United States of America; Azienda Ospedaliero Universitaria Careggi, ITALY

## Abstract

Millions of Americans have been infected with COVID-19 and communities of color have been disproportionately burdened. We investigated the relationship between demographic characteristics and COVID-19 positivity, and comorbidities and severe COVID-19 illness (use of mechanical ventilation and length of stay) within a racial/ethnic minority population. Patients tested for COVID-19 between March 2020 and January 2021 (N = 14171) were 49.9% (n = 7072) female; 50.1% (n = 7104) non-Hispanic Black; 33.2% (n = 4698) Hispanic; and 23.6% (n = 3348) aged 65+. Overall COVID-19 positivity was 16.1% (n = 2286). Compared to females, males were 1.1 times more likely to test positive (p = 0.014). Compared to non-Hispanic Whites, non-Hispanic Black and Hispanic persons were 1.4 (p = 0.003) and 2.4 (p<0.001) times more likely, respectively, to test positive. Compared to persons ages 18–24, the odds of testing positive were statistically significantly higher for every age group except 25–34, and those aged 65+ were 2.8 times more likely to test positive (p<0.001). Adjusted for race, sex, and age, COVID-positive patients with chronic obstructive pulmonary disease were 1.9 times more likely to require a ventilator compared to those without chronic obstructive pulmonary disease (p = 0.001). Length of stay was not statistically significantly associated with any of the comorbidity variables. Our findings emphasize the importance of documenting COVID-19 disparities in marginalized populations.

## Introduction

As of April 5, 2021, there have been over 30.5 million cases of COVID-19 and over 550,000 COVID-19 associated deaths reported in the United States (US) [1]. The clinical manifestation of COVID-19 is variable ranging from asymptomatic to life-threatening [2]. Yet, thus far, there are limited effective treatment options available for COVID-19 [3, 4]. Identifying characteristics that predict severity and subsequent outcome of COVID-19 infection can aid in identifying patients who may benefit from early intervention, monitoring, and vaccination priority, as well as for generating hypotheses for clinical trials [5].

The burden of COVID-19 in the US is not distributed equally. Patients over the age of 65, male, and who have identified as a racial minority are disproportionately represented in morbidity and mortality. Nationwide, patients over 65 represent less than 15% of COVID-19 cases but over 80% of COVID-19 deaths, and males account for 48% of COVID-19 cases but 54% of COVID-19 deaths [6]. Non-Hispanic Black and Hispanic patients experience a higher burden of COVID-19 as compared to non-Hispanic White patients; and are over 2.8 and 3.0 times more likely, respectively, to be hospitalized due to COVID-19 and 1.9 and 2.3 times more likely, respectively, to die from COVID-19 [7, 8]. In 2020, Hispanic individuals between the ages of 25–44 have experienced a 54% increase in deaths, compared to 2019, the largest excess of deaths associated with COVID-19, by age, race, and ethnicity [9]. In Chicago, the distribution and impact of COVID-19 mirrors national trends with those over the age of 65 accounting for 17% of cases but 59% of deaths, men accounting for 48% of cases but 59% of deaths, and non-Hispanic Black individuals representing 30% of the population, but 48% of COVID-19 cases and 41% of COVID-related deaths [8, 10].

In the US, approximately 29% of patients hospitalized with COVID-19 ultimately require intensive care and admission to the intensive care unit (ICU); patients who became critically ill were more likely to be older, male, and have underlying comorbidities [11, 12]. Certain comorbidities, many of which are disproportionately experienced by non-Hispanic Black and Hispanic individuals, may place patients at an increased risk of severe illness from COVID-19, an outcome defined by the Centers for Disease Control (CDC) as “hospitalization, admission to the ICU, intubation or mechanical ventilation, or death” [13, 14]. Several comorbidities have been found to be associated with severe COVID-19, with the most common being hypertension (18.6%), cardiovascular disease (14.4%), and diabetes (11.9%) [15]. In addition, the strongest and most consistent evidence for an association between comorbidities and severe COVID-19 illness is for chronic kidney disease (CKD), chronic obstructive pulmonary disease (COPD), type 2 diabetes, obesity (BMI ≥30 kg/m^2^), smoking and pregnancy [13]. There is some evidence that suggests hypertension, asthma, cerebrovascular disease and a weakened immune system from a different cause may be related to increased risk for severe COVID-19 illness [13]. There is limited evidence that bone marrow transplantation, HIV infection, immune deficiencies, inherited metabolic disorders, liver disease, neurologic conditions, other chronic lung diseases, being overweight (BMI ≥25 but <30 kg/m^2^), thalassemia, and type 1 diabetes are related to severe COVID-19 illness [13].

Globally, population demographics, prevalence of comorbidities, and incidence of COVID-19 vary significantly. This study addresses a gap in the literature by assessing the relationship between patient demographic characteristics and COVID-19 positivity, as well as the relationship between underlying comorbidities and severe COVID-19 illness in an urban safety-net hospital with a primarily racial/ethnic minority patient population.

## Methods

### Setting

Sinai Chicago, Illinois’ largest private safety-net health system, serves the west and southwest sides of Chicago. These under-resourced communities are made up of predominantly minority populations, which suffer a disproportionate burden of chronic disease morbidity and mortality [16].

Sinai Chicago includes Mount Sinai Hospital (MSH), Holy Cross Hospital (HCH), and Schwab Rehabilitation (SRH). MSH is a 319-bed acute care community teaching hospital and an adult Level 1 trauma center; HCH is a 264-bed community hospital; SRH is a 102-bed rehabilitation hospital which serves patients that have experienced spinal cord injuries, stroke, brain injury, amputation, and other musculoskeletal and neurological injuries. MSH and HCH have Emergency Departments (ED) and all three facilities offer a range of inpatient and outpatient services. Sinai Chicago also operates 14 clinics across the system’s service area and includes a range of services including COVID-19 testing.

The majority of persons in the Sinai Chicago service area, as well as patients seen at Sinai Chicago, are un- or under-insured, non-Hispanic Black or Hispanic, and many are Spanish-speaking only [16].

### Study design

We conducted a cross-sectional study of all Sinai Chicago patients 18 years of age and older who were tested for COVID-19 between March 1, 2020 and January 31, 2021 in inpatient and outpatient locations, as well as the ED. The Mount Sinai Hospital Institutional Review Board (FWA#00005088) approved this project (protocol #20–12). Because all of the patients had been discharged at the time of analysis, we were not able to obtain participant consent and the Mount Sinai Hospital Institutional Review Board granted our request for a waiver of HIPAA authorization for research.

### Data collection

Data for this study were abstracted from Meditech, Sinai Chicago’s electronic medical record (EMR).

### Explanatory variables

Explanatory variables included sex, age, race/ethnicity, and patient comorbidities. Sex was a dichotomous variable coded 1 for patients for whom the EMR indicated male sex. Age was a categorical variable coded to groups: 18–24 (referent group), 25–34, 35–44, 45–54, 55–64, and 65+ and calculated as the difference in years between patient date of birth and date of COVID test. Race/ethnicity was a categorical variable coded to groups based on documentation in the EMR: non-Hispanic White (referent group), non-Hispanic Black, Hispanic, non-Hispanic Asian, and Other/Unknown. For patients missing ethnicity data in the EMR, we undertook the following process to recode data. First, of the total records (N = 14171) there were 1234 with Hispanic ethnicity identified in the EMR. For the remaining records (n = 12937), we first applied the surname recode process, which entailed checking the patient’s last name against the 1990 US Census list of the 639 most frequently occurring heavily Hispanic surnames [17] and recoding patients with these last names to Hispanic ethnicity (n = 3149). Second, we manually searched for and evaluated last names with the letters “ez”, “ll”, or “rr” occurring [17] (section 7.1.3) and recoded these to Hispanic ethnicity (n = 107). Finally, for anyone not yet recoded to Hispanic ethnicity, we used the EMR indicator for “patient primary household language is Spanish” to recode to Hispanic ethnicity (n = 208). This brought our total number of records with Hispanic ethnicity to 4698.

Two comorbidity variables were employed: total comorbidities and the Center for Disease Control’s (CDC) three-level comorbidity categorization [18]. Total comorbidities was a categorical variable coded to groups: 0 (referent group), 1, 2, 3–5, and 6–9 and was the sum for patients for whom the EMR indicated an ICD-10 code for cancer, CKD, COPD, cardiovascular disease, obesity, smoking & tobacco use, type 2 diabetes, asthma, hypertension, cerebrovascular disease, HIV, type 1 diabetes, and chronic respiratory conditions (chronic respiratory failure and chronic bronchitis).

We then used the CDC’s recommendations for categorizing these comorbidities into those that have varying levels of evidence for causing severe COVID-19 illness: strongest and most consistent, mixed, and limited evidence. Strongest and most consistent evidence was defined as consistent evidence from multiple small studies or a strong association from a large study. Mixed evidence was defined as multiple studies that reached different conclusions about risk associated with a condition. Limited evidence was defined as consistent evidence from a small number of studies. The strongest evidence category included: cancer, CKD, COPD, cardiovascular disease, obesity, smoking & tobacco use, and type 2 diabetes. The mixed evidence category included: asthma, hypertension, and cerebrovascular disease. The limited evidence category included: HIV, type 1 diabetes, and chronic respiratory conditions [18].

### Outcome variables

The three primary outcome variables of this study were COVID positivity (positivity), COVID-related ventilator use (VENT), and COVID-related length of hospital stay [15].

Positivity was a dichotomous variable coded 1 for patients for whom the EMR indicated a positive COVID test result. Sinai Chicago conducts the SARS-CoV-2 PCR, Rapid, and IgG qualitative tests, which were all included in this analysis. A patient with a positive result for any one or more of those tests was considered positive for COVID-19 (positive). Patients with multiple positive tests were counted only once and for patients with both a positive and negative result, only the positive result was retained in the analysis dataset.

VENT was a dichotomous variable coded 1 for patients for whom the EMR indicated a physician ordered mechanical ventilation of the patient. LOS was a dichotomous variable coded 1 for patients with a length of stay of 3 or more days. A 3-day cutoff point was used as a proxy for severe infection based on the Adaptive COVID-19 Treatment Trial’s (ACTT-1) determination that the benefit of Remdesivir, a therapeutic treatment for COVID-19, is most pronounced among patients who have been hospitalized at least 3 days [19]. Because our sample contained patients tested for COVID-19 at various points during their stay, and in order to ensure our length of stay analysis was most closely related to the COVID-19 infection, we calculated length of stay as the difference between the day the patient was tested for COVID-19 and the day the patient was discharged. This continuous variable was then dichotomized as described above for all patients with non-zero and non-negative values. This was only calculated for patients who were admitted and excluded those who expired during their hospital stay.

### Statistical analysis

Our first aim was to assess whether positivity differed by sex, race/ethnicity, and age. We performed analysis of variance (ANOVA) tests for each demographic characteristic to determine whether differences in positivity were statistically significant between groups.

Our second aim was to assess whether VENT and LOS were associated with comorbidities. We performed logistic regression to calculate odds ratios (OR) for VENT, LOS, total comorbidities, and the strong, mixed, and limited evidence comorbidity variables. Adjusted models include sex, race/ethnicity, and age as control variables. This analysis was restricted to patients with a positive COVID-19 result and additionally, for LOS, the analysis was restricted to patients who did not expire during their COVID-19-related hospital stay.

## Results

### Sample characteristics

A total of 14171 patients were tested for COVID-19 at Sinai Chicago between March 1, 2020 and January 31, 2021 (Table 1). Of those tested, 49.9% (n = 7072) were female; 50.1% (n = 7104) were non-Hispanic Black; 33.2% (n = 4698) were Hispanic; 5.6% (n = 790) were non-Hispanic White; and 23.6% (n = 3348) were over the age of 65.

**Table 1:** Patient COVID-19 tests and results by sex, race, and age.

		COVID tests		Positive COVID result		Negative COVID result		COVID Positivity			
		n	%	n	%	n	%	%	p-value	OR	p
**Patients**		14171	100.0	2286	16.1	11885	83.9	16.1			
**Sex**	Female	7072	49.9	1087	47.6	5985	50.4	15.4	0.014	Ref	Ref
	Male	7099	50.1	1199	52.4	5900	49.6	16.9		1.1	0.014
**Race/Ethnicity**	Non-Hispanic White	790	5.6	81	3.5	709	6.0	10.3	<0.001	Ref	Ref
	Non-Hispanic Black	7104	50.1	997	43.6	6107	51.4	14.0		1.4	0.003
	Hispanic	4698	33.2	1016	44.4	3682	31.0	21.6		2.4	<0.001
	Non-Hispanic Asian	76	0.5	6	0.3	70	0.6	7.9		0.8	0.515
	Other/Unknown	1503	10.6	186	8.1	1317	11.1	12.4		1.2	0.133
**Age**	Age 18–24	1100	7.8	102	4.5	998	8.4	9.3	<0.001	Ref	Ref
	Age 25–34	2381	16.8	269	11.8	2112	17.8	11.3		1.2	0.072
	Age 35–44	2009	14.2	299	13.1	1710	14.4	14.9		1.7	<0.001
	Age 45–54	2380	16.8	389	17.0	1991	16.8	16.3		1.9	<0.001
	Age 55–64	2953	20.8	479	21.0	2474	20.8	16.2		1.9	<0.001
	Age 65+	3348	23.6	748	32.7	2600	21.88	22.3		2.8	<0.001

### Positivity

Of the 14171 patients tested, 2286 (16.1%) had a positive result. Positivity differed by sex (p = 0.014), race/ethnicity (p<0.001), and age (p<0.001) with higher proportions of positive results observed among males (52.4%); Hispanic (44.4%) and non-Hispanic Black persons (43.6%); and persons 65+ (32.7%). Compared to females, males were 1.1 times more likely to test positive for COVID-19 (p = 0.014). Compared to non-Hispanic Whites, non-Hispanic Black and Hispanic persons were 1.4 (p = 0.003) and 2.4 (p<0.001) times more likely, respectively, to test positive for COVID-19. Compared to persons ages 18–24: the odds of testing positive were statistically significantly higher for every age group except 25–34, and those aged 65+ were 2.8 times more likely to test positive for COVID-19 (p<0.001).

### Co-morbidities of positive patients

Among the 2286 positive patients, the most prevalent comorbidities were hypertension (26.8%), type 2 diabetes (23.6%), obesity (10.6%), asthma (7.8%), CKD (7.7%), and COPD (7.3%) (Table 2). Compared to positive males, positive females had a higher prevalence of: COPD, obesity, type 2 diabetes, asthma, hypertension, and chronic respiratory conditions. Positive females also had a higher mean (1.1) total comorbidities compared to positive males (0.8). Compared to positive patients of all other race and ethnicities, non-Hispanic Black positive patients had a higher prevalence of: CKD, COPD, cardiovascular disease, asthma, and chronic respiratory conditions compared to all other race/ethnic groups. Non-Hispanic Black patients (1.3) also had a higher mean total comorbidities compared to positive non-Hispanic White (0.7), and Hispanic (0.7) patients. Compared to positive patients of all other age groups, positive patients 65+ had higher rates of: CKD, COPD, cardiovascular disease, type 2 diabetes, hypertension, cerebrovascular disease, and chronic respiratory conditions. The 65+ age group also had a higher mean (1.3) total comorbidities compared to positive patients of other age groups.

**Table 2:** Comorbidities, ventilator use, and length of stay by result, sex, race, and age.

	**Strong evidence**								
		**Cancer**		**Chronic kidney disease**		**Chronic obstructive pulmonary disease**		**Cardiovascular disease**	
		**n**	**%**	**n**	**%**	**n**	**%**	**n**	**%**
**Full Sample**		149	1.1	832	5.9	1275	9.0	681	4.8
**Positive Test**		18	0.8	177	7.7	168	7.3	151	6.6
**Sex**	Male	9	0.8	100	8.3	87	7.3	79	6.6
	Female	9	0.8	77	7.1	81	7.5	72	6.6
**Race/Ethnicity**	Non-Hispanic Black	7	0.7	106	10.6	123	12.3	94	9.4
	Non-Hispanic White	0	0.0	2	2.5	9	11.1	3	3.7
	Hispanic	10	1.0	58	5.7	25	2.5	43	4.2
	Non-Hispanic Asian	1	16.7	0	0.0	0	0.0	0	0.0
	Other/Unknown	0	0.0	11	5.9	11	5.9	11	5.9
**Age**	Age 18-24	0	0.0	0	0.0	0	0.0	0	0.0
	Age 25-34	0	0.0	7	2.6	2	0.7	5	1.9
	Age 35-44	0	0.0	7	2.3	5	1.7	6	2.0
	Age 45-54	2	0.5	25	6.4	21	5.4	17	4.4
	Age 55-64	7	1.5	40	8.4	47	9.8	32	6.7
	Age 65+	9	1.2	98	13.1	93	12.4	91	12.2
		**Strong evidence**						**Mixed evidence**	
		**Obesity**		**Smoking & Tobacco use**		**Type 2 diabetes**		**Asthma**	
		**n**	**%**	**n**	**%**	**n**	**%**	**n**	**%**
**Full Sample**		1514	10.7	397	2.8	2706	19.1	1455	10.3
**Positive Test**		243	10.6	32	1.4	540	23.6	178	7.8
**Sex**	Male	90	7.5	21	1.8	249	20.8	58	4.8
	Female	153	14.1	11	1.0	291	26.8	120	11.0
**Race/Ethnicity**	Non-Hispanic Black	129	12.9	22	2.2	259	26.0	132	13.2
	Non-Hispanic White	4	4.9	0	0.0	11	13.6	2	2.5
	Hispanic	88	8.7	8	0.8	229	22.5	31	3.1
	Non-Hispanic Asian	1	16.7	1	16.7	4	66.7	0	0.0
	Other/Unknown	21	11.3	1	0.5	37	19.9	13	7.0
**Age**	Age 18-24	3	2.9	1	1.0	1	1.0	7	6.9
	Age 25-34	19	7.1	3	1.1	15	5.6	20	7.4
	Age 35-44	34	11.4	4	1.3	40	13.4	25	8.4
	Age 45-54	47	12.1	3	1.0	96	24.7	28	7.2
	Age 55-64	67	14.0	11	2.3	147	30.7	45	9.4
	Age 65+	73	9.8	10	1.3	241	32.2	53	7.1
		**Mixed evidence**				**Limited evidence**			
		**Hypertension**		**Cerebrovascular disease**		**HIV**		**Type 1 diabetes**	
		**n**	**%**	**n**	**%**	**n**	**%**	**n**	**%**
**Full Sample**		3878	27.4	90	0.6	120	0.8	124	0.9
**Positive Test**		613	26.8	18	0.8	14	0.6	28	1.2
**Sex**	Male	268	22.4	9	0.8	9	0.8	16	1.3
	Female	345	31.7	9	0.8	5	0.5	12	1.1
**Race/Ethnicity**	Non-Hispanic Black	350	35.1	10	1.0	6	0.6	13	1.3
	Non-Hispanic White	22	27.2	1	1.2	0	0.0	0	0.0
	Hispanic	197	19.4	6	0.6	6	0.6	11	1.1
	Non-Hispanic Asian	3	50.0	0	0.0	0	0.0	0	0.0
	Other/Unknown	41	22.0	1	0.5	2	1.1	4	2.2
**Age**	Age 18-24	1	1.0	0	0.0	0	0.0	1	1.0
	Age 25-34	19	7.1	0	0.0	2	0.7	6	2.2
	Age 35-44	46	15.4	0	0.0	6	2.0	6	2.0
	Age 45-54	83	21.3	1	0.3	1	0.3	4	1.0
	Age 55-64	177	37.0	5	1.0	3	0.6	6	1.3
	Age 65+	287	38.4	12	1.6	2	0.3	5	0.7
		**Limited evidence**		**Total Comorbidities**		**Outcomes**			
		**Chronic respiratory conditions**				**Ventilator use**		**Length of stay** **3+ Days**	
		**n**	**%**	**Mean**	**SD**	**n**	**%**	**n**	**%**
**Full Sample**		188	1.3	0.9	1.4	1190	8.4	4373	61.1
**Positive Test**		22	1.0	1.0	1.5	332	1.4	1033	77.0
**Sex**	Male								
	Female	7	0.6	0.8	1.4	206	17.2	612	79.6
**Race/Ethnicity**	Non-Hispanic Black	15	1.4	1.1	1.6	126	11.6	421	73.5
	Non-Hispanic White								
	Hispanic	17	1.7	1.3	1.7	148	14.8	475	78.3
	Non-Hispanic Asian	0	0.0	0.7	1.0	15	18.5	45	78.9
	Other/Unknown	4	0.4	0.7	1.2	148	14.6	429	75.5
**Age**	Age 18-24	0	0.0	1.7	1.0	0	0.0	1	50.0
	Age 25-34	1	0.5	0.8	1.5	21	11.3	83	76.9
	Age 35-44								
	Age 45-54	0	0.0	0.1	0.4	1	1.0	9	34.6
	Age 55-64	1	0.4	0.4	0.9	19	7.1	57	70.4
	Age 65+	1	0.3	0.6	1.1	20	6.7	78	62.4

### Ventilator

During the study period, 332 (1.4%) positive patients at Sinai Chicago required mechanical ventilation (Table 2). Ventilator use was required more frequently among non-Hispanic White patients (18.5%) compared to patients of other race/ethnic groups, male patients (17.2%) compared to female patients, and patients aged 65+ (22.9%) compared to patients in other age groups. In the unadjusted models, ventilator use is statistically significantly associated with two of the strong evidence comorbidities [CKD (OR = 1.5, p = 0.047) and COPD (OR = 2.5, p<0.001)] and one of the limited evidence comorbidities [chronic respiratory conditions (OR = 1.3, p = 0.039)] (Table 3). In the adjusted models, only COPD remained significant such that patients with COVID-19 and COPD were 1.9 times more likely to require a ventilator compared to positive patients without COPD (p = 0.001). We also found that in the unadjusted models, ventilator use is statistically significantly associated with having 3–5 and 6–9 total comorbidities, but once adjusted for our covariates, this association is no longer present and the total number of comorbidities is not associated with a higher odds of ventilator use.

**Table 3:** Multivariate logistic regression odds ratios and p-values for ventilator use and length of stay 3+ days.

		Ventilator use				LOS 3+ days	
		Model 1 (unadjusted)		Model 2 (adjusted)		Model 1 (unadjusted)	
		OR	p	OR	p	OR	p
**Strong Evidence**	Cancer	0.7	0.669				
	Chronic kidney disease	1.5	0.047	1.1	0.531		
	Chronic obstructive pulmonary disease	2.5	<0.001	1.9	0.001		
	Cardiovascular disease	1.4	0.160				
	Obesity	1.2	0.372				
	Smoking & Tobacco use	1.1	0.860				
	Type 2 diabetes	1.2	0.144				
**Mixed Evidence**	Asthma	0.8	0.384				
	Hypertension	1.3	0.065				
	Cerebrovascular disease	0.7	0.669				
**Limited Evidence**	HIV	1.0	0.980				
	Type 1 diabetes	1.3	0.625				
	Chronic respiratory conditions	1.3	0.039	2.3	0.087		
**Total Comorbidities**	0	Ref	Ref	Ref	Ref	Ref	Ref
	1	0.8	0.202	0.7	0.040	1.0	0.880
	2	1.1	0.736	0.9	0.533	0.9	0.522
	3–5	1.5	0.022	1.2	0.386	1.1	0.780
	6–9	2.2	0.036	1.7	0.183	1.2	0.762

### Length of stay

Among positive patients who were admitted to the hospital, stayed at least 1 day, and did not expire during their stay, 1033 (77%) had a length of stay ≥ 3 days. A larger proportion of males (79.6%) and patients aged 65+ (82.7%) had a length of stay ≥ 3 days. Length of Stay was not statistically significantly associated with any comorbidity variables. For total comorbidities, we observed an increased odds with each increment of total comorbidities, but while in the expected direction, these results were not statistically significant. None of the unadjusted models for the strong, mixed, and limited evidence comorbidity variables were statistically significant.

## Discussion

This study demonstrates the significant disparities in COVID-19 positivity by sex, race/ethnicity and age at a large safety-net hospital system on the south and west sides of Chicago. It is also among the first to present findings on the relationship between comorbidities and two outcomes indicative of severe COVID-19 infection: mechanical ventilation and prolonged length of stay. There are a number of key findings from our analysis. First, our results support the vast disparities in COVID-19 positivity among non-Hispanic Black and Hispanic populations. Similar to other findings in urban populations, our study found that non-Hispanic Black and Hispanic patients have a statistically significantly higher odds of positivity (1.5 and 2.5 respectively) [20–22]. Evidence suggests that Hispanic ethnicity is often underreported in healthcare settings, therefore even with our surname recode methodology described above, it is possible this is an underestimate of the impact on the Hispanic community [23]. Some of these disparities can be explained by the long-term disinvestment in these communities which lead to high rates of poverty, poor access to quality healthcare, and distrust of the medical community [24, 25]. Of relevance to the COVID-19 pandemic, non-Hispanic Black and Hispanic persons are more likely to have essential jobs that require them to attend work, and increase their potential for exposure, even when stay-at-home orders are in place [26]. In addition, communities of color are more likely to live in inadequate housing conditions which may lead to crowding and prevent adequate social distancing within multi-generational homes [27].

While non-Hispanic Black and Hispanic patients had a statistically significantly higher odds of positivity compared to patients of other races, they also experienced different rates of positivity from each other. The positivity rate for Hispanic patients was almost twice that of non-Hispanic Black patients (14.0% vs. 21.6%). This may be due to the significant barriers Hispanic patients are more likely to experience to access testing, such as lower rates of insurance coverage, language barriers, and farther distances to testing sites [28].

In addition to racial disparities, our study also explored the impact of specific comorbidities, and total number of comorbidities, on two outcomes of interest: mechanical ventilation and COVID-specific length of stay. Our results suggest that even after adjusting for sex, race/ethnicity, and age, positive patients with a history of COPD have 1.9 times higher odds of requiring mechanical ventilation, an indicator of severe infection. In addition, although not statistically significant, we found that as the total number of comorbidities increases, a patient’s odds of requiring mechanical ventilation also increases.

In summary, our findings emphasize the importance of documenting COVID-19 disparities in marginalized populations to understand the profound impact in these communities and plan for equitable vaccine distribution. Whether or not herd immunity is achieved, all individuals should continue to practice COVID-19 safety precautions such as social distancing and masking. This is particularly true of individuals found to be at the highest risk of severe illness: those with COPD.

### Limitations

While this study only includes data from one health system in Chicago, the sample is unique in that it captures a predominantly racial/ethnic minority population from the largest private safety-net health system in Illinois. Very few studies to date have been able to explore COVID-19 disparities among racial/ethnic minorities, and of note, this is the first study to go a step further and explore the relationship between comorbidities and severe illness as measured by mechanical ventilation and length of stay. In addition, as described above, race/ethnicity data for Hispanic individuals is historically underreported and we faced similar challenges. However, the surname recode methodology utilized in our data cleaning process allowed us to identify almost 3500 additional Hispanic patients, leading to a better estimate of the impact of COVID-19 in this population. The data analyzed for this study did not include individual-level data on risk factors for COVID-19 infection or barriers to testing. However, patients served come from communities characterized by considerable racial inequities in COVID-19 prevalence, mirroring what we see in our results [29]. We selected comorbidities for inclusion in this analysis based on the CDC’s recommendations at the time of analysis. We acknowledge that the literature is constantly changing as we learn more about which comorbidities put patients at higher risk of severe COVID-19 illness. It is also important to note that we did not adjust for having well-controlled comorbidities, either through lifestyle modification or medication. Recent literature suggests that uncontrolled diseases such as hypertension and diabetes are associated with poorer health outcomes [30–32]. Additionally, our comorbidity data may not be comprehensive given that patients may seek care at multiple facilities throughout the city or may not seek care at all. As stated above, the variable for mechanical ventilation was coded based on a physician order for mechanical ventilation in the EMR. We did not do a comprehensive chart review to determine if the patient was truly ventilated. Finally, it is important to acknowledge that treatment protocols changed over the course of our study period; therefore it is possible that patients from the early months of the pandemic may have had worse outcomes than if they had access to treatments that became the standard of care as our understanding evolved.

### Future research

While this study presents strong data to suggest associations between comorbidities and outcomes (mechanical ventilation and length of stay), future research would benefit from including larger datasets across various age ranges, sex, and race/ethnicity to allow us to better understand the disparities within different settings. This study did not include measures of symptomatology which is another opportunity for future research to explore how comorbidities are associated with symptoms of illness such as fever, dyspnea, fatigue, or new loss of taste or smell, as well as likelihood of death as a result of COVID. As of the writing of this paper, three vaccinations have been approved by the Food and Drug Administration for emergency use approval: BioNTech/Pfizer, Moderna, and Johnson & Johnson. While these are just beginning to be widely distributed in the population, future research should include an exploration of how vaccination may impact the progression of disease.

## Supporting information

S1 Dataset (XLSX)
